# Diagnostic value of Revised Baux and Pediatric Baux scores in predicting the outcome of children admitted with burn injuries

**Published:** 2019-08

**Authors:** Farzad Rahmani, Hassan Soleimanpour, Kavous Shahsavari Nia

**Affiliations:** ^*a*^Emergency Medicine Research Team, Assistant Professor of Emergency Medicine, Tabriz University of Medical Sciences, Tabriz, Iran; ^*b*^Cardiovascular Research Center, Professor of Emergency Medicine, Tabriz University of Medical Sciences, Tabriz, Iran.; ^*c*^Road Traffic Injuries Research Center, Associate Professor of Emergency Medicine, Tabriz University of Medical Sciences, Tabriz.

## Abstract

**Background:**

Burn injuries and their consequences are the most important health problems in advanced countries. Prediction of the final outcome of patients with widespread burn injuries in clinical decision making, relief of illness, and optimal allocation of hospital resources for these patients is important and will be helpful in the process of effective triage, implementation of effective therapeutic and surgical interventions, quality control and evaluation, treatment planning, informing the patient's family about the final prognosis and comparing the effectiveness of the therapeutic interventions. Considering that burn injuries are a major concern in children's age groups and require special management and attention, the initial assessment and prediction of the final outcome of children with burn injuries in the implementation of specialized care and reduction of the probability of mortality is an important step for the treatment of these patients. Baux indexes have credible credibility in predicting burn mortality. The purpose of the present study is to determine the value of Revised Baux (R. Baux) and Pediatric Baux (P. Baux) indexes in predicting the probability of mortality, requiring intubation and admission to ICU in a sample of all children in the burning ward of Sina-Tabriz Hospital.

**Methods:**

This descriptive-analytic cross sectional study was carried out in the 6-month period from April to September 2017 in a sample of all children in the burning ward of Sina-Tabriz Hospital. Inclusion criteria include patients less than 12 years of age admitted to the pediatric burn ward and criteria for information exodus are incomplete records. The data were collected in a valid and reliable checklist based on demographic information and specific burn information such as percentage and extent of burn, complications of patients during hospitalization and final outcome. Baux Indexes were calculated for the patients under study. Data were analyzed in SPSS version 17.0. We determined predictive value, sensitivity, specificity and cut-off points of R.Baux and P.Baux for outcome of Children admitted with Burn Iinjuries. P value less than 0.05 is significant.

**Results:**

Of the total of 213 admitted children, 59.60% were male. Most of the children (137; 64.3%) had burns of 2 and 3. 1.6% of children died. The rate of intubation of children with burns in this study was 7.4%. Increasing one point in the R.Baux and P.Baux criteria, despite a constant burn percentage, increased the chance of a patient's death by 43.1 and 42.1, respectively. Increasing one point in the R.Baux and P.Baux criteria, despite the fact that the percentage of burns remained constant, increased the chance of requiring intubation of the patient by 23.1 and 22.1 times. Spearman correlation coefficient between burn percentage and R.Baux and P.Baux was calculated to be 0.928 with P <0.001.

**Conclusions:**

The R.Baux point and P.Baux point are related to the probability of motility and intubation. Also, the percentage of burns is related with the likelihood of need for ICU and correlates with P.Baux and R.Baux. Therefore, using the points obtained and the percentage of body burns, it is possible to reduce the mortality of children with burns with special care and care.

**Keywords:**

Burn, Children, R.Baux, P.Baux

